# Real‐World Outcomes of Combination Therapy With Radium‐223 and Enzalutamide Compared With Enzalutamide Alone in Patients With Metastatic Castration‐Resistant Prostate Cancer

**DOI:** 10.1155/aiu/2359934

**Published:** 2026-02-25

**Authors:** Kenichi Hata, Yuma Goto, Masaki Hashimoto, Yusuke Takahashi, Yuki Takiguchi, Shun Saito, Yuya Iwamoto, Yuki Enei, Keigo Sakanaka, Akira Hisakane, Taisuke Yamazaki, Fumihiko Urabe, Takafumi Yanagisawa, Shunsuke Tsuzuki, Tatsuya Shimomura, Takahiro Kimura, Takanori Tojo, Hirohito Kobayashi

**Affiliations:** ^1^ Department of Urology, Atsugi City Hospital, 1-16-36 Mizuhiki, Atsugi, Kanagawa, 243-8588, Japan, atsugicity-hp.jp; ^2^ Department of Urology, Shonan Keiiku Hospital, 4360 Endo, Fujisawa, Kanagawa, 252-0816, Japan; ^3^ Department of Urology, Jikei University School of Medicine, 3-25-8 Nishishinbashi Minato, Tokyo, 105-8461, Japan, jikei.ac.jp; ^4^ Department of Urology, Tomeiatsugi Hospital, 232 Funako, Atsugi, Kanagawa, 243-8571, Japan; ^5^ Department of Urology, Ebina General Hospital, 4-16-1 Chuo, Ebina, Kanagawa, 243-0432, Japan, ebina.jinai.jp

## Abstract

Radium‐223 and enzalutamide have shown survival benefits in metastatic castration‐resistant prostate cancer, and recent trial data suggest a potential synergy in combination. However, real‐world data on their combined use, especially regarding fracture risk and prior androgen receptor signaling inhibitor exposure, are limited. In this study, we aimed to evaluate the efficacy and safety of adding radium‐223 to enzalutamide compared with enzalutamide alone in patients with bone metastatic castration‐resistant prostate cancer. We retrospectively included patients treated with radium‐223 plus enzalutamide (the combination group) or enzalutamide alone (the enzalutamide group), and compared progression‐free survival, overall survival, prostate‐specific antigen kinetics, alkaline phosphatase kinetics, and treatment‐emergent adverse events. A total of 17 and 18 patients were included in the combination and enzalutamide groups, and the median age and follow‐up duration were 75 years and 23 months, respectively. The median progression‐free survival was significantly longer in the combination group (22 months [95% confidence interval (CI): 16.7–32.4]) than that in the enzalutamide group (5 months [95% CI: 6.3–25.2], *p* = 0.048). However, there was no significant difference in overall survival (combination: not reached [95% CI: 31.1–44.8] vs. enzalutamide: not reached [95% CI: 28.9–56.5], *p* = 0.602). In the combination group, there was no significant difference in progression‐free and overall survival between patients with and without prior androgen receptor signaling inhibitor therapy. Fractures occurred in one (5.9%) and two (11.1%) patients in the combination and enzalutamide groups, respectively. In conclusion, the combination of radium‐223 and enzalutamide did not improve overall survival in patients with metastatic castration‐resistant prostate cancer. However, it significantly prolonged progression‐free survival without increasing the risk of fractures when bone health agents were administered. Furthermore, prior exposure to androgen receptor signaling inhibitors was not associated with oncological outcomes of the combination therapy.

## 1. Introduction

Globally, prostate cancer is currently the fourth most prevalent malignancy and the eighth leading cause of cancer‐related mortality among men [1]. Androgen deprivation therapy (ADT) remains the standard treatment for metastatic prostate cancer [2]. Nonetheless, there have been rapid advancements in the therapeutic landscape in recent years. Despite initial responsiveness to ADT, most patients ultimately experience disease progression, resulting in metastatic castration‐resistant prostate cancer (mCRPC) [3].

Radium‐223 (Ra223) is a bone‐seeking, alpha particle–emitting radiopharmaceutical that mimics calcium and preferentially targets areas of osteoblastic or sclerotic metastasis [4]. Compared with placebo, Ra223 has been shown to significantly prolong overall survival (OS) and the time to the first symptomatic skeletal event [5]. In 2013, it was approved by the U.S. Food and Drug Administration for the treatment of patients with mCRPC, symptomatic bone metastases, and no known visceral metastatic diseases. Its mechanism of action does not overlap with other life‐prolonging agents, and it has emerged as the first bone‐targeted agent with survival benefits in patients with mCRPC.

Combination therapy with Ra223 and other life‐prolonging agents, including androgen receptor signaling inhibitors (ARSIs), chemotherapy, immunotherapy, and checkpoint immunotherapy, has been investigated, given the poor prognosis of mCRPC in patients [6–13]. The data from these exploratory studies suggest that each combination therapy may be effective and safe.

However, a study comparing Ra223 with or without abiraterone (ABI) reported an increased fracture rate (29% vs. 11%) without a survival advantage, especially in patients not receiving bone health agents (BHAs) [14]. Consequently, the Pharmacovigilance Risk Assessment Committee of the European Medicines Agency warned that the safety and efficacy of Ra223 in combination with ARSIs, including enzalutamide (ENZ), remain to be established [14].

Notably, the PEACE‐3 trial, a randomized, multicenter, Phase III study comparing ENZ alone versus ENZ combined with Ra223 in patients with asymptomatic or mildly symptomatic mCRPC, was published [15]. The combination arm showed a significant improvement in radiographic progression‐free survival (PFS) compared with the ENZ arm (the combination arm: 19.4 months vs. the ENZ arm: 16.4 months, hazard ratio [HR], 0.69; 95% confidence interval [CI], 0.54–0.87; *p* = 0.0009). Furthermore, the addition of Ra223 to ENZ resulted in a significant enhancement in OS and time to next systemic treatment, providing robust evidence for the efficacy of this combination in the management of mCRPC. Accordingly, we conducted a retrospective cohort study in patients with mCRPC to evaluate the efficacy and safety of the combination of Ra223 and ENZ compared with ENZ alone in real‐world practice.

## 2. Materials and Methods

### 2.1. Study Design and Population

After receiving institutional review board approval (36‐005[12104]), we retrospectively analyzed 35 consecutive patients with mCRPC with bone metastases treated with ENZ, with or without Ra223, between January 2018 and May 2024 at Atsugi City Hospital, Kanagawa, Japan. Given the retrospective nature of the study, informed consent was obtained through an opt‐out process, allowing patients or their representatives to decline participation after being provided with detailed study information. This study adhered to all applicable regulations regarding patient confidentiality and data protection. All patients had histologically confirmed adenocarcinoma and castration‐resistant disease with bone metastasis, as evidenced by increased prostate‐specific antigen (PSA) levels as well as radiographic or clinical progression during ADT. Eligible patients were divided into two cohorts: those who received Ra223 plus ENZ (the combination group) and those who received ENZ alone (the ENZ group). with or without BHAs. Attending physicians and patients jointly decided between combination therapy and ENZ monotherapy based on patient preference. The exclusion criteria were malignant lymphadenopathy with a short‐axis diameter > 3 cm, a history of visceral metastases, and treatment with external beam radiation during the study period. Lymph node metastases with a short‐axis diameter of 3 cm or less were not considered exclusionary.

### 2.2. Treatment Procedure

Ra223 was intravenously administered at a dose of 55 kBq/kg at 4‐week intervals for up to six doses. ENZ was orally administered at a daily dose of 160 mg. In the combination group, all patients received ENZ within 1 month before or after Ra223 administration. Except for one patient, all patients in the combination group received BHAs. In contrast, the decision to administer BHAs to patients in the ENZ group was left to the discretion of the attending physician. The patients continued ENZ treatment until radiographic and/or clinical progression was observed. Furthermore, ADT (i.e., luteinizing hormone–releasing hormone agonist, antagonist, or surgical castration) was continued until optimal supportive care was provided. The treatment sequence following disease progression was determined at the discretion of each physician.

### 2.3. Patient Characteristics

We manually extracted the following data from institutional medical records: patient characteristics which included age, follow‐up period, Eastern Cooperative Oncology Group Performance Status (PS), prior therapy for prostate cancer, treatment line of Ra223, and concurrent use of BHAs. Additionally, we collected data regarding oncological characteristics, including PSA at diagnosis, tumor stage, Gleason score, time to CRPC, PSA and alkaline phosphatase (ALP) levels at treatment initiation, presence of lymph node metastases, and extent of disease (EOD) score.

### 2.4. Follow‐Up

Patients were generally followed up every 1–3 months for a check‐up, which included history taking, physical examination, routine blood tests, and serum chemistry. Thoracoabdominopelvic computed tomography and/or bone scintigraphy were performed every 6–12 months and when clinically indicated. PSA and serum hematological examinations were performed at baseline, monthly during the Ra223 period, and at least every 3 months thereafter. PFS was defined based on the Prostate Cancer Clinical Trials Working Group 2 criteria: castrate serum testosterone < 50 ng/dL or 1.7 nmol/L, plus biochemical progression comprising three consecutive increases in PSA levels at 1‐week intervals resulting in two 50% increases over the nadir and a PSA > 2 ng/mL, radiological progression characterized by the appearance of new bone lesions on bone scan or a new soft tissue lesion in accordance with the Evaluation Criteria in Solid Tumors Version 1.1. [16], and/or death.

The PSA90 and ALP30 responses were indicated as ≥ 90% PSA decline or ≥ 30% ALP decline from baseline, respectively, with confirmation through another measurement after ≥ 4 weeks.

Treatment‐emergent adverse events (TEAEs) and fractures were identified by reviewing the medical records from the date of treatment initiation to the observation period.

### 2.5. Statistical Analysis

Continuous variables are presented as medians and interquartile ranges (IQRs), while categorical variables are presented as numbers and percentages. Between‐group comparisons of patient characteristics were performed using independent *t*‐tests for numerical data (age, follow‐up period, initial PSA level, time to CRPC, and baseline PSA and ALP levels) and the chi‐square test/Fisher’s exact test for categorical data (PS, tumor stage, Gleason score, EOD score, prior therapies, treatment line in CRPC, and concurrent use of BHAs). PFS and OS were calculated using the Kaplan–Meier method and compared using the log‐rank test for each prognostic variable. HRs and 95% CIs were derived using Cox proportional hazards models.

All statistical analyses were performed using BellCurve statistical software (BellCurve Inc., Tokyo, Japan). Statistical significance was set at a two‐sided *p* value < 0.05.

## 3. Results

### 3.1. Patient Characteristics

Table 1 shows the patient characteristics in each group. Among the 35 eligible patients, 17 and 18 were assigned to the combination and ENZ groups, respectively, between January 2018 and May 2024. The median age of the patients at treatment initiation was 75 years (IQR, 73–77 years) and 76 years (IQR, 70.5–77.8 years) in the combination and ENZ groups, respectively. With the data cutoff date set at May 31, 2024, there was no significant between‐group difference in the median follow‐up period (combination group: 30 months, IQR: 18–39 months vs. ENZ group: 11.5 months, IQR: 6–36.5 months; *p* = 0.477). There were no significant between‐group differences in most baseline demographic and clinical characteristics, except for the median time to CRPC, EOD score, and concurrent use of BHAs (*p* = 0.027, 0.001, and 0.018, respectively).

**TABLE 1:** Baseline characteristics of patients receiving study treatment.

	**Total**	**Combination group**	**ENZ group**	**p** **value**

No. of patients, number	35	17	18	
Age (years), median (IQR)	75 (72–77.5)	75 (73–77)	76 (70.5–77.8)	0.984
Follow‐up period (months), median (IQR)	23 (7.5–40)	30 (18–39)	11.5 (6–36.5)	0.477
ECOG‐PS, number (%)				0.486
0‐1	34 (97.1)	16 (94.1)	18 (100)	
2	1 (2.9)	1 (5.9)	0 (0)	
Initial PSA (ng/mL), median (IQR)	250 (31.1–984)	250 (29–1110)	212.5 (34.7–8895)	0.483
Tumor stage at diagnosis, number (%)				0.625
T1‐2	5 (14.3)	3 (17.6)	2 (11.1)	
T3‐4	26 (74.3)	10 (58.8)	16 (88.9)	
Missing	4 (11.4)	4 (23.5)	0 (0)	
Gleason score at diagnosis, number (%)				1.0
< 8	4 (11.4)	2 (11.8)	2 (11.1)	
≥ 8	31 (88.6)	15 (88.2)	16 (88.9)	
Time to CRPC (months), median (IQR)	23 (12.5–49)	18 (9–28)	41.5 (19–76)	0.027
Baseline PSA (ng/mL), median (IQR)	5.5 (2.7–20.6)	2.8 (2.1–8.9)	8.1 (4.8–32.4)	0.208
Baseline ALP (U/L), median (IQR)	196 (99.5–255.5)	130 (79–222)	213.5 (141.8–270.3)	0.886
EOD score, number (%)				0.001
1	17 (48.6)	3 (17.6)	14 (77.8)	
2, 3	18 (51.4)	14 (82.4)	4 (22.2)	
Lymph node metastases, number (%)	9 (25.7)	2 (11.8)	7 (38.9)	0.12
Prior therapies, number (%)				
Abiraterone/prednisone	16 (45.7)	6 (35.3)	10 (55.6)	
Docetaxel	13 (37.1)	4 (23.5)	9 (50)	
Cabazitaxel	2 (5.7)	0 (0)	2 (11.1)	
Treatment line in CRPC, number (%)				0.289
First line	23 (65.7)	13 (76.5)	10 (55.6)	
Later line	12 (34.3)	4 (23.5)	8 (44.4)	
Concurrent use of BHAs, number (%)				0.018
Use	26 (74.3)	16 (94.1)	10 (55.6)	

Regarding the EOD score, 82.4% of the patients in the combination group had an EOD of 2 or 3, whereas 77.8% of those in the ENZ group had an EOD of 1. All patients in the combination group received the study treatment as either first‐line or second‐line therapy. In contrast, in the ENZ group, 44.4% of the patients received it as a later therapy. However, no significant between‐group differences were observed. Accordingly, the ENZ group had a higher proportion of patients who received docetaxel or cabazitaxel prior to treatment, whereas none of the patients in the combination group received cabazitaxel. The ENZ group had significantly fewer patients who received BHAs than those in the combination group.

### 3.2. Oncologic Outcomes

Progression occurred in nine (52.9%) and 15 (83.3%) patients in the combination and ENZ groups, respectively. As shown in Figure 1(a), the median PFS was significantly longer in the combination group than in the ENZ group (combination: 22 months [95% CI: 16.7–32.4] vs. ENZ: 5 months [95% CI: 6.3–25.2], *p* = 0.048). However, there was no significant between‐group difference in OS (combination: not reached [95% CI: 31.1–44.8] vs. ENZ: not reached [95% CI: 28.9–56.5], *p* = 0.602, Figure 1(b)).

Figure 1Kaplan–Meier curves depicting progression‐free survival (a) and overall survival (b) in patients with metastatic castration‐resistant prostate cancer treated with the combination therapy of radium‐223 and enzalutamide versus enzalutamide alone. Abbreviations: PFS, progression‐free survival; OS, overall survival; Ra223, radium‐223; ENZ, enzalutamide; CI, confidence interval; NR, not reached.

**Figure:**
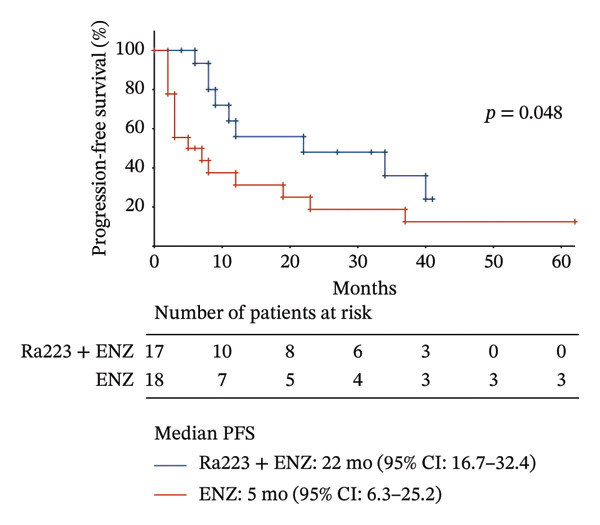
(a)

**Figure:**
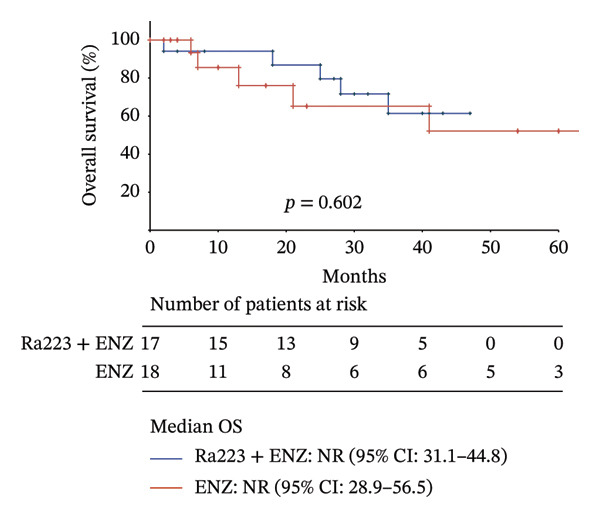
(b)

### 3.3. Oncological Outcomes in Relation to Prior ARSI Use in the Combination Group

In the combination group, six patients previously received ARSIs therapy, while 11 did not. Notably, there were no significant differences in the PFS or OS between patients with and without prior ARSI therapy (Figures 2(a) and 2(b)).

Figure 2Kaplan–Meier curves depicting progression‐free survival (a) and overall survival (b) within the combination therapy cohort, which was stratified by prior use of androgen receptor signaling inhibitors. Abbreviations: PFS, progression‐free survival; OS, overall survival; Ra223, radium‐223; ENZ, enzalutamide; CI, confidence interval; NR, not reached.

**Figure:**
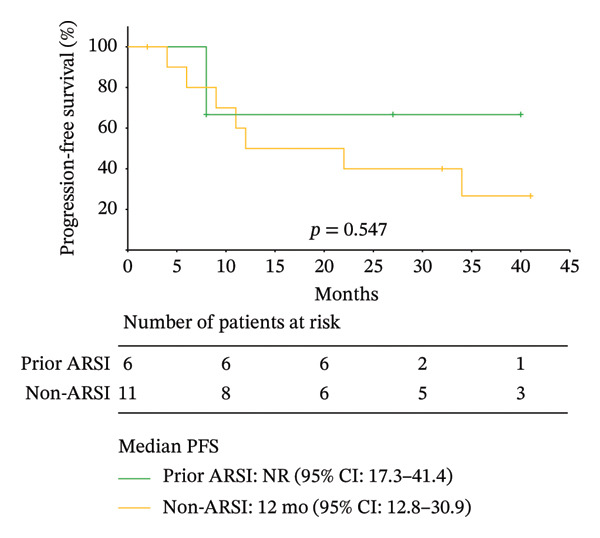
(a)

**Figure:**
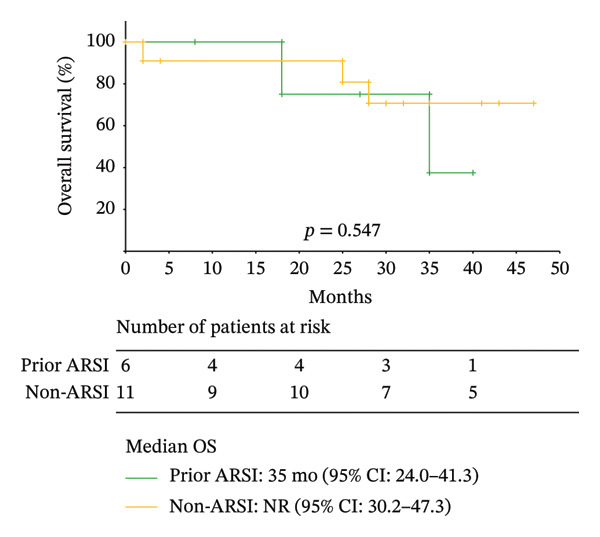
(b)

### 3.4. PSA and ALP Responses

As shown in Figures 3(a) and 3(b), there was no significant between‐group difference in the PSA90 (combination: six patients, 35.3%; ENZ: six patients, 33.3%; *p* = 0.092). Notably, ALP levels decreased in all patients in the combination group (Figure 4(a)). However, there was no significant between‐group difference in the ALP response (*p* = 0.122, Figures 4(a) and 4(b)). Seven and four patients in the combination and ENZ groups, respectively, achieved ALP30.

Figure 3Waterfall plot of percental PSA change after administration of the study treatment (relative to the baseline), with bar coloring based on the biochemical response. The red and blue bars represent increases and decreases in PSA levels, respectively. (a) Radium‐223 and enzalutamide combination group. (b) Enzalutamide monotherapy group. Abbreviation: PSA, prostate‐specific antigen.

**Figure:**
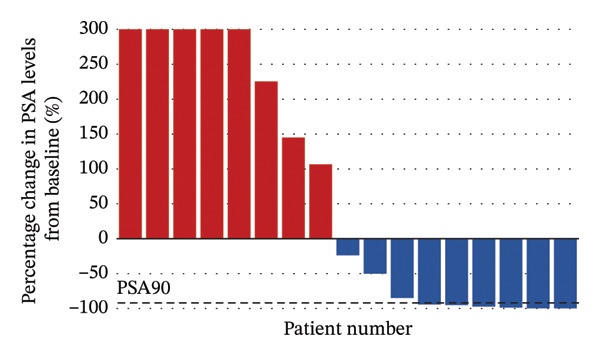
(a)

**Figure:**
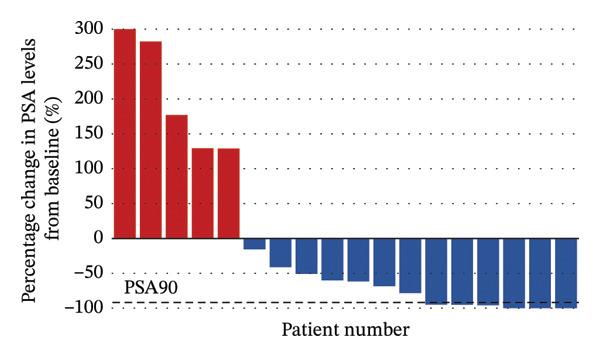
(b)

Figure 4Waterfall plot of percental ALP change after administration of the study treatment (relative to the baseline), with bar coloring according to the biochemical response. The red and blue bars represent increases and decreases in ALP levels, respectively. (a) Radium‐223 and enzalutamide combination group. (b) Enzalutamide monotherapy group. Abbreviations: ALP, alkaline phosphatase; Ra223, radium‐223; ENZ, enzalutamide.

**Figure:**
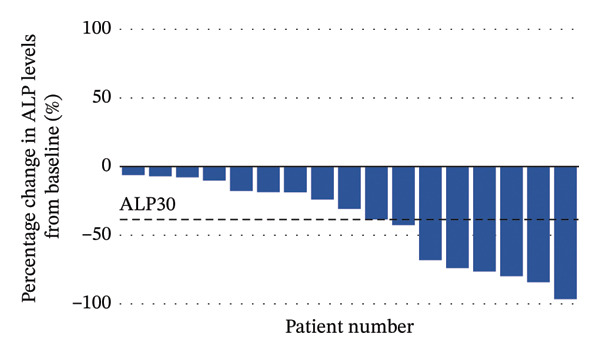
(a)

**Figure:**
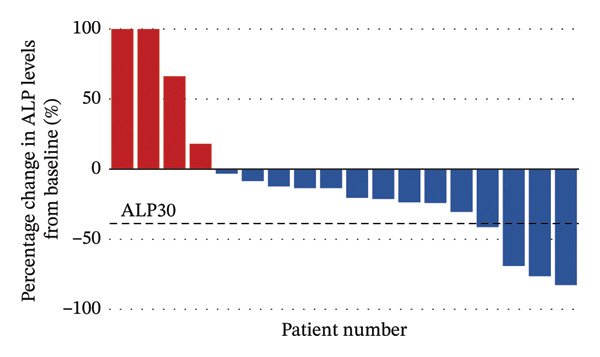
(b)

### 3.5. TEAEs and Fractures

Table 2 summarizes the incidence of TEAEs and fractures during the study period. Notably, the combination group had a lower incidence of fatigue than the ENZ group. The most frequent TEAE in the combination therapy group was appetite loss. The incidence of Grade 3 and 4 AEs was low in both groups.

**Table 2:** Safety profile reported during treatment.

	**Combination group (*n* = 17)**		**ENZ group (*n* = 18)**	
	**All grades**	**≥ Grade3**	**All grades**	**≥ Grade3**

	*N* (%)	*N* (%)	*N* (%)	*N* (%)
Fatigue	1 (5.9)		9 (50)	1 (5.6)
Headache			1 (5.6)	
Dysgeusia	1 (5.9)		1 (5.6)	
Epigastralgia	1 (5.9)		1 (5.6)	
Constipation	1 (5.9)		1 (5.6)	
Diarrhea	1 (5.9)			
Appetite loss	4 (23.5)	1 (5.9)	3 (16.7)	1 (5.6)
Epistaxis	2 (11.8)		1 (5.6)	
Dyspnea	1 (5.9)		1 (5.6)	
Numbness	1 (5.9)		4 (22.2)	
Musculoskeletal pain	3 (17.6)	2 (11.8)		
Arthralgia			2 (11.1)	
Edema			1 (5.6)	
Pollakiuria	1 (5.9)	1 (5.9)	2 (11.1)	
Hot flush	1 (5.9)		1 (5.6)	
Hypertension			2 (11.1)	
AST/ALT increased			1 (5.6)	
Neutropenia	1 (5.9)	1 (5.9)		
Thrombocytopenia			1 (5.6)	
Hypocalcemia			1 (5.6)	
Fracture (vertebral compression)	1 (5.9)		2 (11.1)	

Fractures occurred in one (5.9%) and two (11.1%) patients in the combination and enzalutamide groups, respectively. All three patients had vertebral compression fractures. Among them, one patient in each group received concurrent BHAs .

## 4. Discussion

Our findings indicated that the combination of Ra223 and ENZ in patients with mCRPC allowed a significantly better PFS than ENZ alone in real‐world practice. However, there was no significant between‐group difference in OS. Additionally, prior use of ARSIs was not associated with oncological outcomes in the combination group. Ra223 mainly induces double‐stranded DNA breaks that cause a potent and highly localized cytotoxic effect in the target areas [4, 17, 18]. ENZ has a greater relative binding affinity to the androgen receptor than first‐generation ARSIs, which reduces the efficiency of its nuclear translocation and impairs DNA binding to androgen‐response elements and recruitment of coactivators [19]. The combination of Ra223 and ENZ inhibits LNCaP cell proliferation and exhibits synergistic efficacy in vitro [20]. In vivo, combination therapy has demonstrated enhanced antitumor efficacy, as indicated by serum PSA levels in the intratibial LNCaP model, as well as a decrease in the total area of tumor‐induced abnormal bone [20]. However, the efficacy of the combination therapy in real‐world practice remains unclear.

Tombal et al. reported that Ra223 with ENZ and BHAs as first‐line treatment for mCRPC increases radiographic PFS, and OS benefit was observed in the interim analysis [15]. Our study represents the first report on oncological outcomes of this combination therapy in a real‐world setting, including approximately one‐third of patients treated in later‐line settings. Compared to the PEACE‐3 trial, our study cohort was approximately 5 years older, had a lower median PSA level, and a higher median ALP level. In the combination group, our study demonstrated a median PFS of 22 months, whereas the PEACE‐3 trial reported a median radiographic PFS of 19.4 months. While direct comparison is not possible due to differences in study design and patient characteristics, the results suggest comparable efficacy. Several studies have compared Ra223 monotherapy with combination therapy of Ra223 with ABI or ENZ. A post hoc exploratory analysis of the ALSYMPCA study showed that the concomitant use of ABI or ENZ improved OS [21]. In real‐world settings, Kim et al. and Wakita et al. reported that concomitant use of ABI or ENZ improved PFS compared with Ra223 monotherapy [22, 23]. However, these studies and those of Zhao et al. reported no difference in OS [24], which is consistent with our findings. However, the control agent was Ra223, which differs from our study agents. Additionally, there were no significant between‐group differences in the PSA response, ALP response, or the proportion of patients with PSA90 or ALP30 responses. This suggests that while Ra223 and ENZ combination therapy has a positive impact on PFS, it may not translate into prolonged OS or an improved biochemical response. Further studies are warranted to elucidate the underlying factors contributing to these observations and to identify potential biomarkers that may predict the response to combination therapy.

In this study, prior ARSI therapy was not associated with oncological outcomes in the combination therapy group. Given that doublet and triplet therapies are currently established as standard treatments for mCSPC, many patients with mCRPC may have already been treated with ARSIs [25–29]. Collectively, our findings suggest that, even after treatment with ARSIs, combination therapy with Ra223 and ENZ remains effective.

In the PEACE‐3 trial, hypertension was the most frequently reported TEAE, while fatigue ranked third in frequency [15]. Notably, the incidence of fatigue, a TEAE associated with ENZ, was significantly lower in the combination group than in the ENZ group [30, 31]. Although the exact cause remains unclear, Ra223 may alleviate ENZ‐induced fatigue. This effect may be partially attributed to the higher proportion of later‐line therapy in the ENZ group. In the PEACE‐3 trial, Gillessen et al. compared the incidence of fractures between Ra223 combined with ENZ for first‐line mCRPC and ENZ alone. In that trial, the rate of BHA use was 97%, given the report from the ERA223 trial indicating a high fracture rate in the study and control arms [32]. In our study, fractures were more frequent in the ENZ group; however, the incidence was lower in both groups compared with that among BHA nonusers in the PEACE‐3 trial (15.6% and 37.1% in the ENZ and combination arms, respectively) [32]. This finding may be attributed to the lower frequency of BHA use in the ENZ group. Taken together, these results highlight the importance of administering BHAs in combination therapy, including ARSI monotherapy for mCRPC [14, 32].

This study had some limitations. First, this study had a small sample size and a relatively short observation period. Second, this was a retrospective, single‐center study; additionally, drug selection was generally at the discretion of the attending physicians. Third, compared to the ENZ group, the combination group exhibited a shorter time to CRPC and higher EOD scores. This suggests that patients in the combination group had more aggressive disease and a higher tumor burden, which may have influenced the oncological outcomes. Despite these constraints, we believe our findings offer valuable preliminary evidence that may provide insights into designing future multicenter studies and support clinical decision‐making in real‐world settings.

## 5. Conclusions

In this real‐world retrospective analysis, the combination of Ra223 and ENZ did not demonstrate a significant improvement in OS; however, it significantly prolonged PFS without increasing fracture risk. Additionally, prior use of ARSIs was not associated with the oncological outcomes of the combination therapy. These findings suggest that Ra223 plus ENZ may be a viable treatment option for patients with mCRPC, including those previously treated with ARSIs. Nonetheless, the interpretation of these findings is limited by the small sample size and the inherent selection bias characteristic of retrospective studies. Further prospective studies with larger cohorts are required to validate these findings.

## Funding

No funding was received for this study.

## Ethics Statement

This study was approved by the Institutional Review Board of the Jikei University School of Medicine (no. R4‐7), including permission for publication. Informed consent was obtained using an IBR‐approved opt‐out method.

## Conflicts of Interest

Takahiro Kimura has received consulting or advisory fees from Astellas, Bayer, Janssen, and Sanofi. The other authors declare no conflicts of interest.

## Data Availability

Data supporting the findings of this study are available from the corresponding author upon reasonable request.
